# Ultrasound-assisted deep eutectic solvent extraction of polyphenols from peach leaves and their compositional characterization

**DOI:** 10.3389/fnut.2026.1937286

**Published:** 2026-08-20

**Authors:** Qingsheng Zhou, Yalong Liang, Benguo Liu

**Affiliations:** 1 School of Geography and Tourism, Zhengzhou Normal University, Zhengzhou, China; 2 School of Food Science, Henan Institute of Science and Technology, Xinxiang, China

**Keywords:** antioxidant activity, deep eutectic solvents, peach leaves, polyphenols, ultrasound-assisted extraction

## Abstract

This work reports the extraction of polyphenols from peach leaves via ultrasound-assisted deep eutectic solvent extraction (UAE-DES) coupled with subsequent resin-based purification, along with comprehensive characterization of the resulting phenolic profile. Screening of various DES formulations identified the choline chloride–lactic acid system as the most effective for this purpose. Based on single-factor experiments and Box–Behnken response surface design, the optimal UAE-DES extraction parameters were determined as follows: liquid-to-solid ratio of 26.61 mL/g, ultrasonic temperature of 40 °C, choline chloride-to-lactic acid molar ratio of 1:3.715, water content of 20%, and sonication time of 10 min. Under these optimal settings, the total phenolic content (TPC) attained 16.79 mg gallic acid equivalents (GAE)/g, closely approaching the model-predicted value of 16.92 mg GAE/g. The crude extract was subsequently purified using AB-8 macroporous resin, and Fourier transform infrared spectroscopy (FTIR) analysis confirmed the presence of functional groups characteristic of phenolic and flavonoid structures. UPLC–MS/MS profiling further identified 25 distinct phenolic compounds, with flavonol glycosides representing the dominant category, accounting for 91.07% of the total quantified polyphenols. Among these, quercetin-3-O-glucoside (50.75%), kaempferol-3-O-glucoside (20.71%), and rutin (19.61%) were identified as the most abundant components. *in vitro* antioxidant evaluation revealed that the purified peach leaf polyphenols possessed potent concentration-dependent radical-scavenging capacity against both ABTS^+^ and DPPH radicals. Collectively, these results demonstrate that the UAE-DES/resin purification approach offers a green and effective means of recovering polyphenols from peach leaves, thereby providing a scientific foundation for the valorization of peach leaf resources and the production of natural antioxidant agents.

## 1 Introduction

Peach (*Prunus persica* L.), a member of the Rosaceae family, is an economically important stone fruit tree cultivated worldwide. China is the world's largest producer of peaches, accounting for more than half of global production, generating large quantities of pruning residues in the form of pruned branches and leaves. However, current disposal practices for this pruning waste are predominantly limited to abandonment, composting, or incineration, resulting in low resource conversion efficiency and underutilized economic value. In recent years, agricultural by-products have gained increasing attention as valuable sources of natural bioactive ingredients. Phenolic acids, flavonoids, and other secondary metabolites enriched in fruit tree leaves have been recognized as important reservoirs of functional natural products. Therefore, developing green and efficient approaches for the utilization of functional components from peach leaves is of great significance for extending the peach industry value chain and promoting the circular utilization of agricultural waste.

Previous studies have demonstrated that peach leaves are rich in polyphenolic compounds, including chlorogenic acid, anthocyanins, and flavonoids, which exhibit remarkable free radical-scavenging, antioxidant, and potential anti-inflammatory properties (1–4). Additionally, they contain dietary fiber, vitamins, and minerals, providing a basis for the development of functional ingredients. Currently, leaf-derived products such as mulberry leaf tea, persimmon leaf tea, and loquat leaf tea have been widely commercialized in the functional beverage market due to their high phenolic content (5, 6). In traditional Chinese medicine, peach leaves have been used for internal medicinal purposes for over two millennia. Nevertheless, research on peach leaves has primarily focused on pharmacological evaluations, with limited attention paid to the efficient and green preparation of their polyphenolic components. Particularly in the context of rapid advances in clean extraction technologies, comprehensive studies remain scarce, which hinders the high-value conversion of peach leaf resources into natural antioxidants and functional teas.

The efficient and green preparation of plant polyphenols is a prerequisite for their functional utilization. Although classic organic solvent-based extraction techniques provide acceptable extraction performance, they suffer from disadvantages including high solvent usage, considerable environmental impact, and safety hazards. Deep eutectic solvents (DESs), due to their low toxicity, biodegradability, and strong solvation capacity, have gained attention as eco-friendly substitutes for extracting bioactive phytochemicals from plant matrices (7, 8). In particular, choline chloride-based natural DES systems can enhance the solubility of target compounds through hydrogen-bonding networks, thereby facilitating cell wall disruption and the release of active ingredients (9, 10). However, the high viscosity of DESs restricts mass transfer efficiency, necessitating auxiliary intensification strategies. Ultrasound-assisted extraction (UAE) can enhance diffusion and cell rupture through cavitation effects (11, 12). The UAE-DES combination strategy has been shown to exert considerable synergistic effects during the environmentally friendly recovery of phenolics from diverse plant materials and is now considered a key technological approach (13).

However, crude DES extracts often contain co-extracted impurities, such as sugars, proteins, and pigments, which require further enrichment and purification to improve the purity and bioactivity of the target compounds (14). AB-8 macroporous adsorption resin, owing to its favorable adsorption selectivity and desorption performance, is widely employed for the separation and purification of plant polyphenols (15). Modern analytical platforms, including ultra-performance liquid chromatography–tandem mass spectrometry (UPLC–MS/MS), allow for comprehensive profiling of phenolic constituents, thereby supporting the identification of bioactive components and the exploration of structure–function relationships (16). However, studies integrating UAE-DES extraction, resin purification, and compositional characterization of peach leaf polyphenols are still lacking, and the comprehensive phenolic profiles as well as the antioxidant efficacy of these extracts have not been systematically evaluated.

In this context, the present study aimed to utilize peach leaves as raw materials to extract polyphenols via UAE-DES, optimize the extraction parameters using response surface methodology (RSM), and perform enrichment and purification using AB-8 resin. Furthermore, the chemical composition of purified peach leaf polyphenols was characterized by UPLC–MS/MS, and their antioxidant activities were evaluated. This integrated strategy establishes a green and efficient preparation system for peach leaf polyphenols, enabling their extraction, purification, chemical composition characterization, and antioxidant activity evaluation, thereby providing scientific support for the high-value utilization of peach leaf resources.

## 2 Materials and methods

### 2.1 Materials and chemicals

Peach (*Prunus persica*) leaves, obtained from Qixutang Co., Ltd. (Baoding, China), were processed into a homogeneous powder using a laboratory mill, sieved through a 100-mesh screen, and hermetically sealed in dry containers at ambient temperature until further analysis. Gallic acid and choline chloride were the products of Aladdin (Shanghai, China). AB-8 resin and Folin-Ciocalteu reagent were from Yuanye Biotechnology Co., Ltd. (Shanghai, China). Analytical-grade reagents were used for all other chemicals.

### 2.2 Preparation of deep eutectic solvents (DESs)

A series of deep eutectic solvents (DESs) were formulated following the heating–stirring approach described by Liu et al. (17). In each formulation, choline chloride served as the hydrogen bond acceptor (HBA), while a panel of five compounds–ethylene glycol, lactic acid, glycerol, urea, and glucose–were individually employed as hydrogen bond donors (HBDs). The molar ratio of HBA to HBD was fixed at 1:2 (mol/mol). The weighed reagents were transferred into screw-capped glass bottles, followed by the addition of deionized water to adjust the water content to 20% (w/w). The mixtures were heated at 80 °C under constant stirring until clear, homogeneous liquids, free of any precipitate, formed. The resulting DESs were then cooled to ambient temperature and stored for later use.

### 2.3 Determination of total phenolic content (TPC)

TPC measurements were performed using the Folin–Ciocalteu colorimetric assay with minor adjustments to the protocol established by Islamcevic et al. (18). In this procedure, 300-μL portions of gallic acid reference solutions spanning 0.2–1.0 mg/mL were combined with 9.7 mL of distilled water and 0.5 mL of the Folin–Ciocalteu reagent. After a 5 min reaction step, 5 mL of 5% (w/v) sodium carbonate was introduced, and distilled water was added to bring the final volume to 25 mL. The solutions were then incubated for 90 min in the dark at room temperature, after which the absorbances were recorded at 765 nm. A calibration graph was generated by correlating absorbance readings with gallic acid standard concentrations. Identical treatment was given to sample solutions, with TPC results ultimately reported as mg GAE per gram of sample.

### 2.4 Single-factor experiments

A series of single-factor trials were undertaken to evaluate how different extraction parameters affect the TPC yield from peach leaves. Throughout these experiments, the DES water content was kept constant at 20% (w/w), while the following variables were assessed separately: liquid-to-solid ratio (15–35 mL/g), choline chloride-to-lactic acid molar ratio (1:1–1:5), sonication duration (10–75 min), and ultrasonic temperature (15 °C−75 °C). After each extraction run, the resulting blends were centrifuged (10,000 × g, 20 min), and the clear upper phases were recovered for subsequent TPC quantification according to the protocol outlined in **Section 2.3**.

### 2.5 Response surface methodology (RSM)

Optimization of the peach leaf polyphenol extraction conditions was carried out via a Box–Behnken experimental design, with the ranges for each factor determined from earlier single-factor trials. A quadratic polynomial model was constructed using Design-Expert software (version 13.0, Stat-Ease Inc., Minneapolis, MN, USA) to characterize the relationship between the independent variables-liquid-to-solid ratio (A, mL/g), ultrasonic temperature (B, °C), and choline chloride-to-lactic acid molar ratio (C)—and the dependent response, TPC yield (mg GAE/g) (Table 1). The design matrix comprised 17 experimental runs covering three factors at three levels, including five replicates at the center point. Multiple regression of the acquired data yielded a second-order polynomial equation, which was subsequently employed to identify the optimal extraction settings.

**Table 1:** Response surface factors and levels.

Factor levels	Liquid to solid ratio (mL/g)	Ultrasonic temperature (°C)	Molar ratio
	A	B	C
−1	25	15	1:2
0	30	30	1:3
1	35	45	1:4

### 2.6 Purification of peach leaf polyphenols

The AB-8 macroporous adsorption resin was preconditioned according to the method of Wang et al. (19), with slight procedural adjustments. In brief, the resin was placed in absolute ethanol for 24 h to achieve complete swelling, after which it was thoroughly washed with ultrapure water. Subsequently, the resin was subjected to sequential soaking in 5% (w/v) hydrochloric acid and 5% (w/v) sodium hydroxide, each for 4 h. Following each acid or base treatment, the resin was rinsed with deionized water to neutrality and finally conserved in ethanol until use. The conditioned resin was then packed into a glass column by the wet-packing approach, with the flow rate regulated via a constant-flow pump. The crude peach leaf polyphenol extract was applied onto the column for dynamic adsorption. Once adsorption reached saturation, the column was washed successively with deionized water and 20% (v/v) ethanol at 1.5 mL/min to remove unwanted constituents, followed by elution with 70% (v/v) ethanol. The effluent was gathered as 10 mL portions, concentrated under vacuum to eliminate ethanol, and lyophilized to afford purified peach leaf polyphenols (20). In this study, the AB-8 resin was used only for a single purification cycle, and no regeneration or reuse procedure was performed.

### 2.7 FTIR analysis

For FTIR analysis, the purified peach leaf polyphenol sample was intimately mixed with KBr in a 1:100 weight ratio, pulverized to a fine homogeneous powder, and subsequently compacted into transparent discs. Spectral data were collected over the 4000–400 cm^−^^1^ range on an FTIR spectrometer, with 4 cm^−^^1^ resolution and 32 co-added scans per spectrum.

### 2.8 UPLC–MS/MS analysis of polyphenol composition

For the identification and quantification of individual phenolic constituents, exactly weighed portions of the purified peach leaf polyphenol extract were taken up in 80% aqueous methanol (v/v) containing 0.2% ascorbic acid (w/v) and subjected to 30 min of sonication at ambient temperature. After centrifugation (12,000 × g, 10 min), the resulting supernatant was collected, suitably diluted, and injected into a UHPLC system connected to a Q Exactive Orbitrap high-resolution mass spectrometer (Thermo Fisher Scientific, Waltham, MA, USA). Chromatographic separation was achieved on a Waters HSS T3 column (2.1 × 50 mm, 1.8 μm particle diameter) with a mobile phase composed of 0.1% formic acid in ultrapure water (solvent A) and acetonitrile (solvent B), operated under gradient elution at a flow rate of 0.30 mL/min. The column was thermostated at 40 °C, and the injection volume was 2 μL per run. Mass spectra were obtained in negative-ion electrospray ionization mode (ESI^−^) over the m/z 100–900 range. The identification was accomplished by matching retention times and accurate masses against those of authentic reference standards, with an acceptable mass deviation of less than 10 ppm. Quantification was carried out using external standard calibration. TraceFinder™ software (Thermo Fisher Scientific, Waltham, MA, USA) was utilized to process all raw data, encompassing peak detection, retention-time alignment, component identification, and peak-area integration (21).

### 2.9 Determination of antioxidant activity

The DPPH radical-scavenging activity of the purified extracts was assessed following a published procedure with minor modifications (22). Briefly, 500-μL aliquots of sample solutions prepared at varying concentrations were mixed with an equivalent volume (500 μL) of 0.2 mmol/L DPPH dissolved in absolute ethanol. The reaction mixtures were kept in darkness at ambient temperature for 30 min, after which the absorbances were read at 517 nm. The percentage of scavenging activity was determined from Equation (1):


$$\begin{array}{c} \text{DPPH }\left(\text{\%}\right)=\left(1-\frac{A_{1}-A_{2}}{A_{3}}\right)\times 100\% \end{array}$$ (1)

where A_1_ denotes the absorbance recorded for the sample–DPPH mixture; A_2_ corresponds to the sample–ethanol blank, and A_3_ represents the control (water–DPPH).

The ABTS^+^ radical-scavenging activity of the samples was evaluated according to the protocol described in reference (22), with minor modifications. The ABTS^+^ working reagent was generated by combining equal volumes of 7.4 mmol/L ABTS and 2.6 mmol/L potassium persulfate (K_2_S_2_O_8_), allowing the mixture to react in the dark for 14 h, followed by dilution with absolute ethanol until the absorbance reached 0.70 ± 0.02 at 734 nm. For antioxidant assessment, 200 μL aliquots of each sample were mixed with 800 μL of the ABTS^+^ working solution and kept in the dark for 6 min, after which the absorbance was read at 734 nm. The ABTS^+^ scavenging percentage was then computed using Equation (2):


$$\begin{array}{c} \text{ABTS }\left(\text{\%}\right)=\left(1-\frac{A_{1}-A_{2}}{A_{3}}\right)\times 100\% \end{array}$$ (2)

where A_1_ refers to the absorbance of the sample–ABTS^+^ mixture, A_2_ indicates the sample–ethanol blank, and A_3_ is the blank control (water–ABTS^+^).

### 2.10 Statistical analysis

All experimental measurements were carried out in triplicate, and the resulting data were expressed as arithmetic means along with their standard deviations. Intergroup comparisons were assessed by one-way ANOVA using IBM SPSS Statistics 21.0, coupled with Duncan's *post-hoc* test for multiple comparisons at the 95% confidence level (*p* < 0.05). Origin 2024 was used to generate all graphical representations of the data, whereas the response surface methodology (RSM) experimental layout and subsequent model-fitting procedures were carried out using Design-Expert 13.0.

## 3 Results and discussion

### 3.1 Screening of the optimal deep eutectic solvent system

Five binary deep eutectic solvents (DESs) containing 20% (w/w) water were successfully prepared and appeared as homogeneous, transparent liquids at room temperature. The extraction yields of peach leaf polyphenols obtained using different DES systems are presented in Table 2. The choice of hydrogen bond donor (HBD) markedly affected the polyphenol extraction yield from peach leaves. Among the DES formulations tested, the choline chloride–lactic acid system gave the highest TPC yield, reaching 14.04 ± 0.31 mg GAE/g, followed by the choline chloride–urea system, whereas DESs based on ethylene glycol, glycerol, and glucose showed considerably lower extraction efficiencies. The superior performance of the lactic acid-based DES can be primarily attributed to its favorable physicochemical properties. Organic acid-based DESs generally possess relatively low viscosity and appropriate polarity, which facilitate solvent penetration into plant tissues and enhance mass transfer during extraction. In addition, the mildly acidic environment may promote the release of bound phenolic compounds by disrupting interactions between phenolics and cell wall components, thereby increasing the extractable phenolic fraction. Similar findings have been reported by Dai et al. (23), who demonstrated that organic acid-based DESs improve the solubility of natural products through the synergistic effects of polarity and acidity. Likewise, Zainal-Abidin et al. (24) summarized that organic acid-based DESs exhibit superior extraction performance for food bioactive compounds owing to their ability to disrupt plant cell wall structures and promote the release of intracellular metabolites. In contrast, DESs prepared with polyols or sugars generally exhibit higher viscosities, which increase mass transfer resistance and hinder the diffusion of phenolic compounds from plant tissues into the extraction medium, resulting in reduced extraction efficiency (25). The relatively high extraction yield obtained with the choline chloride–urea DES may be attributed to the strong hydrogen-bonding capability of urea, which enhances the solubility of polyphenols through intermolecular hydrogen-bond interactions. This explanation is consistent with the mechanism proposed by Abbott et al. (26). In summary, the choline chloride–lactic acid system outperformed all other DES formulations examined in terms of extraction yield and was consequently chosen for subsequent optimization studies.

**Table 2:** Effect of DES system on TPC yield.

DES system	Molar ratio	TPC yield (mg GAE/g)
Choline chloride–ethylene glycol	1:2	8.50 ± 0.89
Choline chloride–glycerol	1:2	6.48 ± 0.23
Choline chloride–lactic acid	1:2	14.04 ± 0.31
Choline chloride–urea	1:2	12.26 ± 0.43
Choline chloride–glucose	1:2	7.25 ± 0.19

### 3.2 Single-factor experimental results

#### 3.2.1 Effect of the liquid-to-solid ratio on the extraction of peach leaf polyphenols

**Figure 1:**
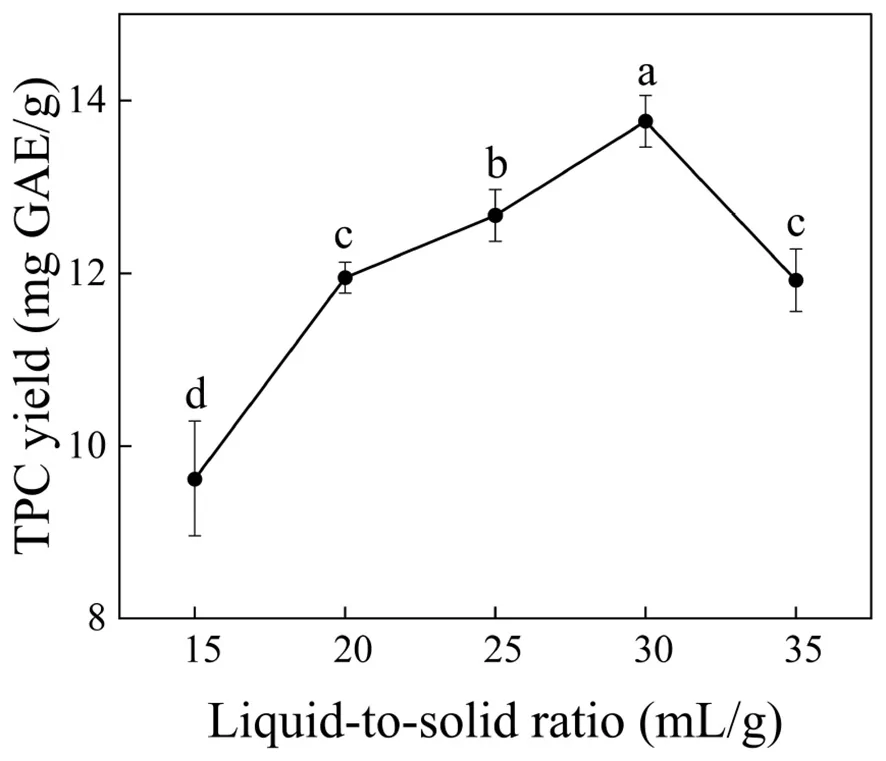
Effect of liquid-to-solid ratio on the extraction yield of peach leaf polyphenols. Values are expressed as mean ± standard deviation (SD) (*n* = 3). Different lowercase letters indicate significant differences among treatments (*p* < 0.05).

With the choline chloride–lactic acid (ChCl–Lac) system served as the most effective DES, the influence of varying liquid-to-solid ratios on polyphenol yield from peach leaves was subsequently examined. Figure 1 illustrates that the TPC yield rose sharply as the liquid-to-solid ratio increased, peaked within a certain intermediate zone, and then progressively fell with further rises, suggesting that an optimum ratio existed for the extraction process.

Such behavior is explicable in terms of mass-transfer dynamics and solvent–matrix interactions. Under low-ratio conditions, the restricted solvent volume diminished the interfacial area accessible to the plant material, leading to poor mass transfer and hampered phenolic diffusion. Elevating the liquid-to-solid ratio strengthened the concentration differential between the solid matrix and the extracting phase while concurrently lowering the system's apparent viscosity, thus promoting both phenolic dissolution and diffusional transport. Consistent findings were documented by Antonio et al. (27), who highlighted the liquid-to-solid ratio as a pivotal determinant governing mass-transfer efficiency during the recovery of bioactive phytochemicals. Nevertheless, exceeding the optimal ratio range caused a reduction in TPC yield. This reduction may be attributed to the dilution of the extracted compounds, which weakens the concentration gradient driving mass transfer. Moreover, excessive solvent volume may attenuate the intensity of ultrasonic cavitation, thereby decreasing cell disruption and limiting the release of intracellular polyphenols (28). In addition, excessive dilution may alter the hydrogen-bonding network of the DES, reducing its affinity for polyphenolic compounds. Similar trends have also been reported for the extraction of plant polyphenols using DESs, where extraction efficiency increased only within an appropriate liquid-to-solid ratio range before reaching a plateau or decreasing (24). A liquid-to-solid ratio of 30 mL/g afforded the highest extraction yield under the present experimental conditions. Consequently, the range of 25–35 mL/g was adopted for the subsequent RSM optimization.

#### 3.2.2 Effect of the choline chloride-to-lactic acid molar ratio on the extraction of peach leaf polyphenols

**Figure 2:**
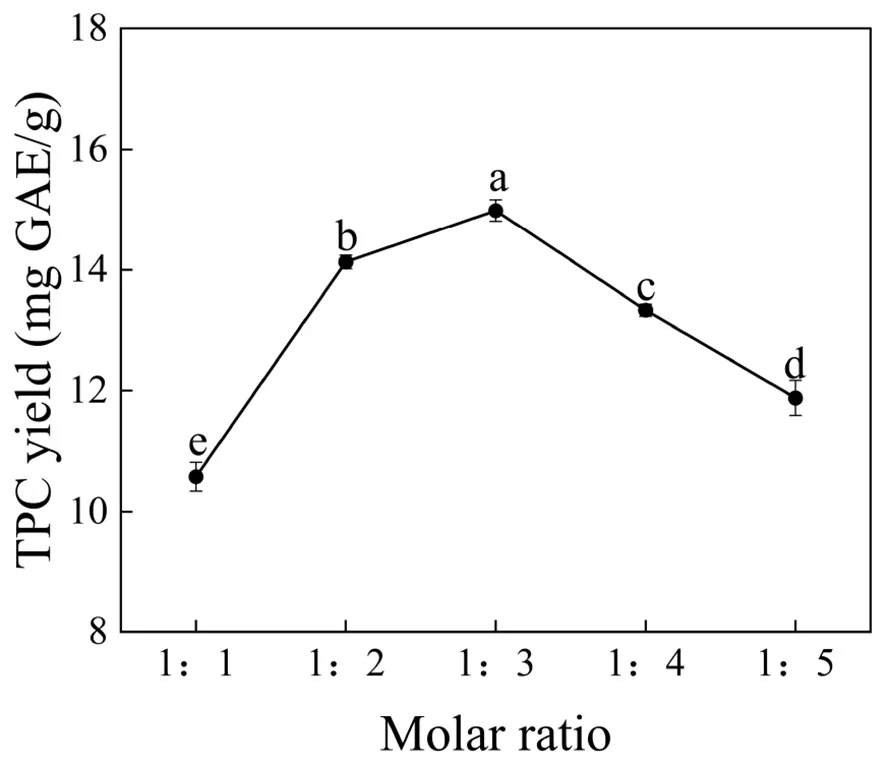
Effect of the molar ratio of choline chloride to lactic acid on the extraction yield of peach leaf polyphenols. Values are expressed as mean ± standard deviation (SD) (*n* = 3). Different lowercase letters indicate significant differences among treatments (*p* < 0.05).

With the optimal DES system identified as choline chloride–lactic acid (ChCl–Lac), the impact of varying the HBA-to-HBD molar ratio on polyphenol extraction from peach leaves was explored further, with all other operational parameters held constant. Figure 2 reveals that the TPC yield rose progressively as the lactic acid content increased, maximized when the ChCl-to-lactic acid ratio reached 1:3, and then declined steadily upon further addition of lactic acid. These observations demonstrate that the relative proportion of HBA to HBD exerts a critical influence on the extraction efficiency of the DES system.

This phenomenon can be attributed to alterations in the hydrogen-bonding architecture and the physical–chemical characteristics of the DES. The HBA-to-HBD molar ratio directly modulates the population and stability of intermolecular hydrogen bonds, which in turn affects solvent polarity, viscosity, and its overall binding affinity toward phenolic species. At relatively low lactic acid contents (e.g., ChCl = 1:1), the insufficient amount of hydrogen bond donor limited the formation of a stable hydrogen-bonding network, resulting in reduced solvent–solute interactions and poor extraction efficiency. As the proportion of lactic acid increased, a more stable hydrogen-bonding network was established, providing an appropriate solvent polarity for the dissolution of polyphenols. In parallel, the mildly acidic conditions contributed to the breakdown of plant cell wall architectures and facilitated the liberation of cell wall-bound phenolic compounds, thus improving extraction yields. Comparable mechanistic observations were documented by Rashid et al. and Moni Bottu et al. (29, 30). However, when the lactic acid proportion exceeded the optimal ratio (≥1:4), the TPC yield decreased. Excessive HBD may disturb the optimal hydrogen-bonding equilibrium within the DES, resulting in a less favorable solvent microstructure and weaker solvent–solute interactions. In addition, increased acidity may promote the degradation or structural transformation of certain phenolic compounds, while also altering the solvent microenvironment and its extraction selectivity (31). Previous studies have likewise demonstrated that the extraction efficiency of DESs is closely associated with the stability of their hydrogen-bonding networks, and deviations from the optimal molar ratio generally lead to reduced extraction performance (32). In summary, the HBA-to-HBD molar ratio exerted a marked influence on the TPC yield through modulation of the DES hydrogen-bonding architecture and its physicochemical characteristics. Under the present experimental conditions, maximum yield was attained when the ChCl-to-lactic acid ratio was set at 1:3. Consequently, a molar ratio span of 1:2–1:4 was chosen for the ensuing response surface optimization studies.

#### 3.2.3 Effect of ultrasonic extraction time on the extraction of peach leaf polyphenols

**Figure 3:**
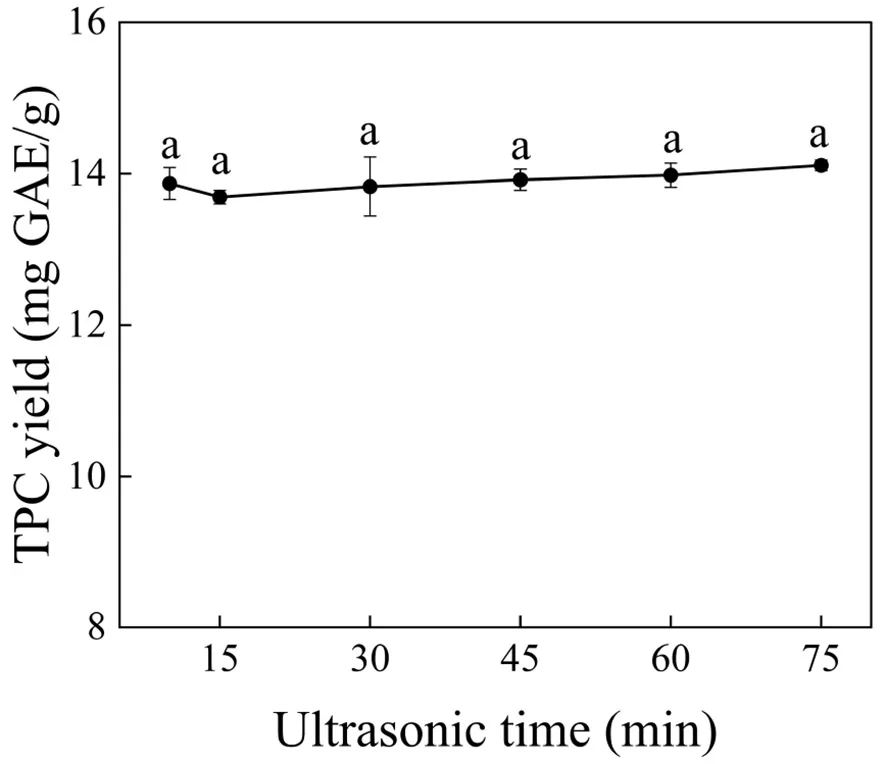
Effect of ultrasonic time on the extraction yield of peach leaf polyphenols. Values are expressed as mean ± standard deviation (SD) (*n* = 3). Different lowercase letters indicate significant differences among treatments (*p* < 0.05).

The influence of sonication duration (10–75 min) on polyphenol recovery from peach leaves was examined while maintaining all other parameters constant. Figure 3 demonstrates that the TPC yield remained relatively stable across the entire time window examined, with no statistically meaningful variations detected among the different treatment durations (*p* > 0.05). These findings suggest that extending the sonication period beyond 10 min did not appreciably affect extraction performance under the conditions tested. The rapid establishment of extraction equilibrium is attributable to the cavitational effects generated during probe sonication. The violent collapse of cavitation bubbles gives rise to localized micro-jets and shockwaves that efficiently break down plant cell wall structures, consequently facilitating solvent infiltration and improving the diffusional transport of intracellular phenolic species into the surrounding extraction fluid (33). In the present study, the TPC yield reached a stable level within 10 min, suggesting that the ChCl–Lac DES possessed excellent solvent accessibility and affinity for peach leaf polyphenols, enabling rapid extraction of the target compounds. Further extension of the ultrasonic treatment did not significantly improve the extraction efficiency, probably because the concentration gradient between the plant matrix and the extraction solvent gradually decreased until mass transfer equilibrium was reached. Moreover, prolonged ultrasonic treatment may generate reactive oxygen species and localized heating, which could induce the oxidation or degradation of certain phenolic compounds, thereby offsetting any potential improvement in extraction efficiency. Comparable trends have been noted in previous studies employing ultrasound-assisted extraction of plant polyphenols, wherein extending the treatment duration past the equilibrium stage yielded negligible further gains. In view of both extraction yield and energy expenditure, a 10-min sonication period was adopted for all subsequent experiments.

#### 3.2.4 Effect of ultrasonic temperature on the extraction of peach leaf polyphenols

**Figure 4:**
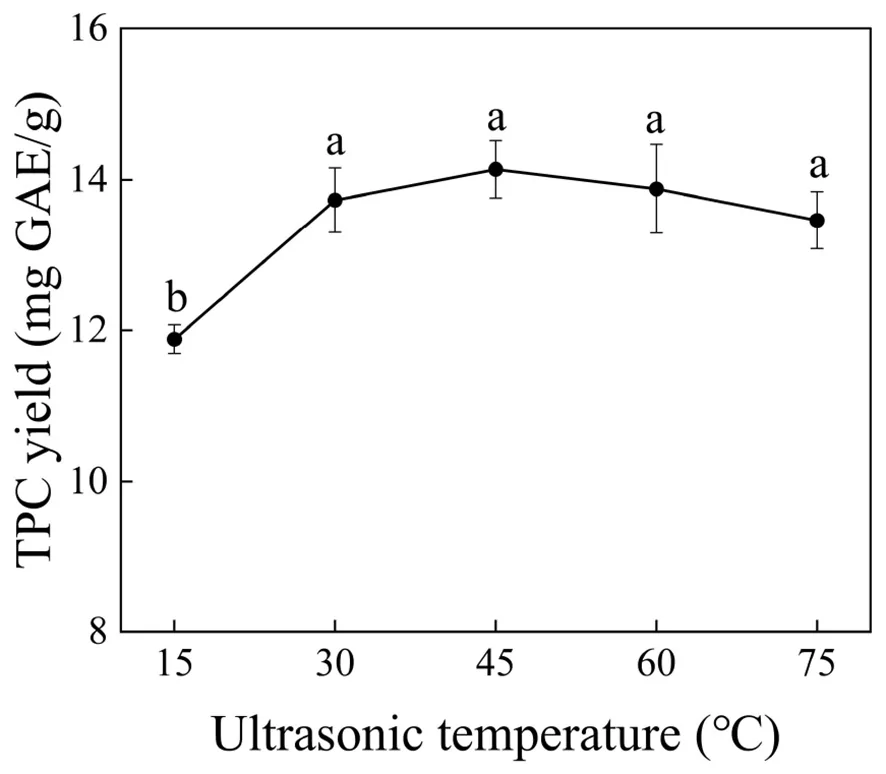
Effect of extraction temperature on the extraction yield of peach leaf polyphenols. Values are expressed as mean ± standard deviation (SD) (*n* = 3). Different lowercase letters indicate significant differences among treatments (*p* < 0.05).

The influence of ultrasonic temperature (ranging from 15 °C to 75 °C) on the yield of polyphenols from peach leaves was examined, with all remaining extraction variables held fixed. Figure 4 illustrates that the TPC yield rose progressively as the temperature was elevated, peaked at approximately 45 °C, and then showed a modest reduction upon further temperature elevation. Statistical analysis indicated that no significant differences were observed among the extraction temperatures ranging from 30 °C to 75 °C (*p* > 0.05), whereas the TPC yields obtained within this temperature range were significantly higher than those at 15 °C, suggesting that moderate heating effectively enhanced the extraction process.

The influence of temperature on the UAE-DES system can be attributed to the combined effects of enhanced mass transfer and ultrasonic cavitation. Increasing the extraction temperature reduced the viscosity of the deep eutectic solvent, thereby improving its fluidity, penetration into plant tissues, and diffusion of phenolic compounds. Furthermore, moderate temperatures facilitated the disruption of plant cell architecture, thereby releasing intracellular phenolic compounds. Similar temperature-dependent enhancement has been widely reported for ultrasonic-assisted extraction of plant bioactive compounds (34, 35). However, further increases in temperature did not result in a significant improvement in TPC yield. At elevated temperatures, the intensity of ultrasonic cavitation may decrease because vapor-filled cavitation bubbles collapse less violently, thereby reducing the mechanical disruption of plant tissues. Although higher temperatures improve solvent diffusivity, the weakened cavitation effect may offset these advantages. Furthermore, excessive temperatures may promote the oxidation or thermal degradation of heat-sensitive phenolic compounds, leading to a reduction in extraction efficiency (36). Overall, extraction temperature exerted a dual effect on the UAE-DES process. Moderate heating enhanced solvent mass transfer and polyphenol dissolution, whereas excessive temperatures weakened ultrasonic cavitation and might adversely affect the stability of phenolic compounds. Considering the comparable TPC yields obtained at 30 °C and 45 °C, together with energy consumption and the stability of the extracted compounds, the ultrasonic temperature range of 15 °C−45 °C was selected for the subsequent RSM optimization.

### 3.3 Response surface optimization

**Figure 5:**
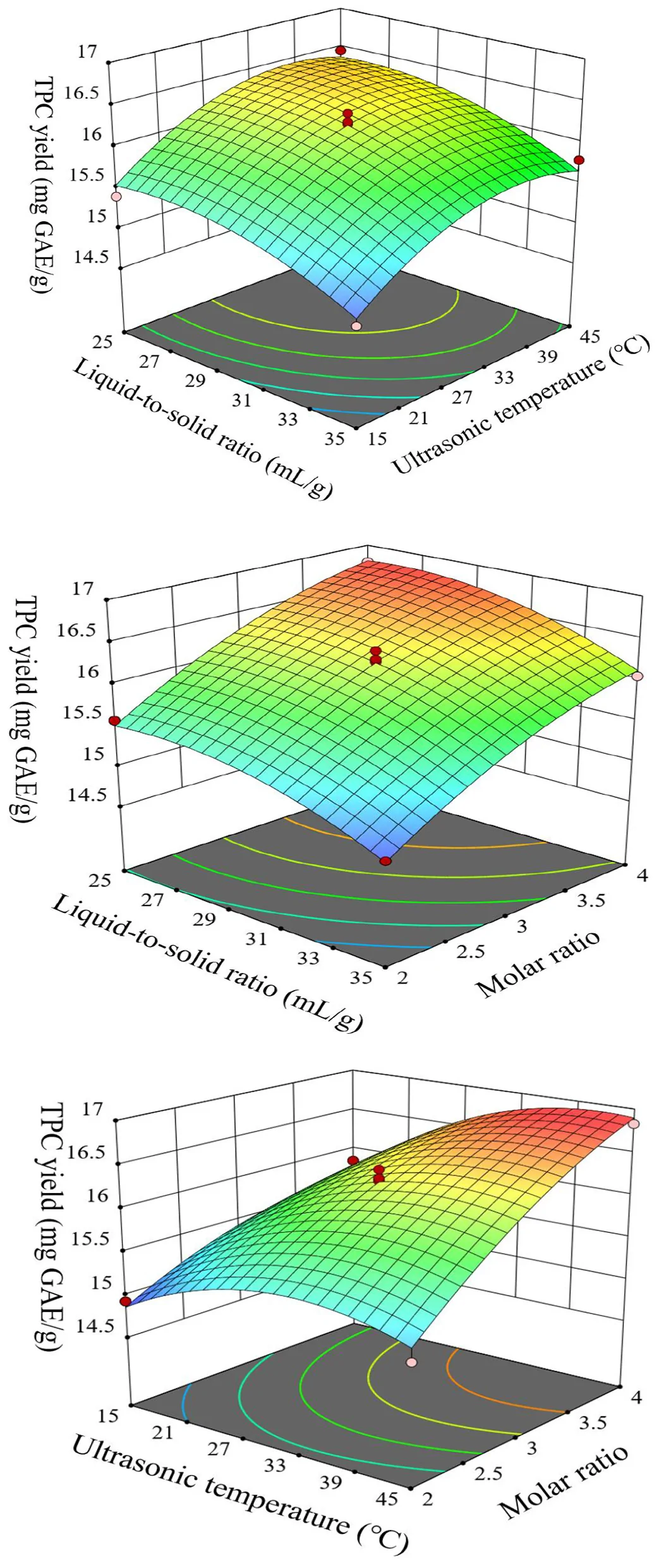
Effect of the interactions between variables on the extraction yield of peach leaf polyphenols.

To optimize the extraction conditions, a three-factor, three-level Box–Behnken design was adopted, with 17 individual runs performed in total. The independent input variables included the liquid-to-solid ratio (25–35 mL/g), ultrasonic temperature (15 °C−45 °C), and choline chloride-to-lactic acid molar ratio (1:2–1:4), while TPC yield (Y) was specified as the output response. Fixed parameters throughout the experiments included the water content (20%, w/w) and sonication time (10 min). The full experimental scheme is summarized in Table 3, and the corresponding three-dimensional response surfaces are depicted in Figure 5.

**Table 3:** Experimental design and RSM results.

RSM experiment					ANOVA					
Run	A (mL/g)	B (°C)	C	Y (mgGAE/g)	Source	Sum of squares	df	Mean square	*F*-value	*p*-value
1	35 (1)	30 (0)	1:2 (−1)	14.90	Model	6.51	9	0.7237	31.52	< 0.0001
2	25 (−1)	30 (0)	1:4 (1)	16.78	A	0.8450	1	0.8450	36.80	0.0005
3	30 (0)	30 (0)	1:3 (0)	16.18	B	1.19	1	1.19	51.64	0.0002
4	35 (1)	30 (0)	1:4 (1)	16.05	C	3.20	1	3.20	139.38	< 0.0001
5	25 (−1)	30 (0)	1:2 (−1)	15.57	AB	0.0036	1	0.0036	0.1568	0.7039
6	30 (0)	30 (0)	1:3 (0)	16.28	AC	0.0009	1	0.0009	0.0392	0.8487
7	35 (1)	45 (1)	1:3 (0)	15.79	BC	0.1849	1	0.1849	8.05	0.0251
8	30 (0)	30 (0)	1:3 (0)	16.38	A^2^	0.2160	1	0.2160	9.41	0.0181
9	30 (0)	30 (0)	1:3 (0)	16.25	B^2^	0.6290	1	0.6290	27.39	0.0012
10	30 (0)	15 (−1)	1:4 (1)	15.85	C^2^	0.1465	1	0.1465	6.38	0.0395
11	30 (0)	45 (1)	1:2 (−1)	15.05	Residual	0.1607	7	0.0230		
12	25 (−1)	15 (−1)	1:3 (0)	15.40	Lack of fit	0.1163	3	0.0388	3.48	0.1296
13	30 (0)	30 (0)	1:3 (0)	16.10	Credibility analysis of the regression equations					
14	25 (−1)	45 (1)	1:3 (0)	16.45	Std. dev.	0.1515	R-squared	0.9759		
15	35 (1)	15 (−1)	1:3 (0)	14.86	Mean	15.86	Adj. R-squared	0.9450		
16	30 (0)	15 (−1)	1:2 (−1)	14.93	Second-order polynomial equation					
17	30 (0)	45 (1)	1:4 (1)	16.83	*Y* = 16.24 – 0.325A + 0.385B + 0.6325C – 0.030AB – 0.015AC + 0.215BC – 0.2265A^2^ – 0.3865B^2^ – 0.1865C^2^					

Analysis of variance (ANOVA) demonstrated that the quadratic model reached statistical significance at *p* < 0.0001, with R^2^ = 0.9759 and adjusted R^2^ = 0.9450, demonstrating excellent correspondence between the experimental observations and the model-derived estimates. The lack-of-fit test was not significant (*p* > 0.05), further confirming that the model appropriately represented the relationship linking the independent factors to the response and possessed satisfactory predictive ability.

Among the three factors examined, the choline chloride-to-lactic acid molar ratio (C) exerted the greatest influence on TPC yield, followed in descending order by the liquid-to-solid ratio (A) and ultrasonic temperature (B). This hierarchy implies that DES composition was the primary determinant of polyphenol solubility, whereas the extraction parameters predominantly governed mass transfer efficiency. Regarding interactive effects, only the BC term (ultrasonic temperature × molar ratio) reached statistical significance (*p* < 0.05), whereas the AB (liquid-to-solid ratio × ultrasonic temperature) and AC (liquid-to-solid ratio × molar ratio) interactions did not. As depicted in Figure 5, the BC surface exhibited considerable curvature, indicating that the temperature effect on extraction was contingent upon the DES molar ratio: raising the temperature enhanced polyphenol release when the molar ratio was near-optimal, yet this benefit subsided as the ratio deviated from this range. By contrast, the relatively planar AB and AC surfaces pointed to negligible synergy between the liquid-to-solid ratio and the other two variables.

All three quadratic coefficients (A^2^, B^2^, and C^2^) reached statistical significance (*p* < 0.05), suggesting that each of the extraction variables possessed an optimal operational window. The three-dimensional response surfaces displayed clear parabolic curvature, which aligned well with the single-factor experimental outcomes and further corroborated the dependability of both the experimental setup and the regression model.

According to the regression model, the optimal extraction conditions were predicted to be a liquid-to-solid ratio of 26.61 mL/g, an ultrasonic temperature of 39.994 °C, and a choline chloride-to-lactic acid molar ratio of 1:3.715, with a predicted TPC yield of 16.92 mg GAE/g. For practical implementation, the ultrasonic temperature was rounded to 40 °C, while the remaining parameters were maintained. Validation experiments performed under these conditions produced a TPC yield of 16.79 mg GAE/g. This result was in good agreement with the predicted value, confirming the reliability and predictive capability of the response surface model.

Overall, the response surface analysis demonstrated that the extraction performance of peach leaf polyphenols was jointly regulated by the composition of the deep eutectic solvent and the extraction parameters. The choline chloride-to-lactic acid molar ratio proved to be the most influential factor among those tested, whereas the liquid-to-solid ratio and ultrasonic temperature played secondary roles by mediating mass transfer during the extraction process. The developed regression model exhibited excellent goodness of fit and predictive performance, providing a reliable basis for the green and efficient extraction of peach leaf polyphenols.

### 3.4 Characterization of peach leaf polyphenols

#### 3.4.1 FTIR analysis

**Figure 6:**
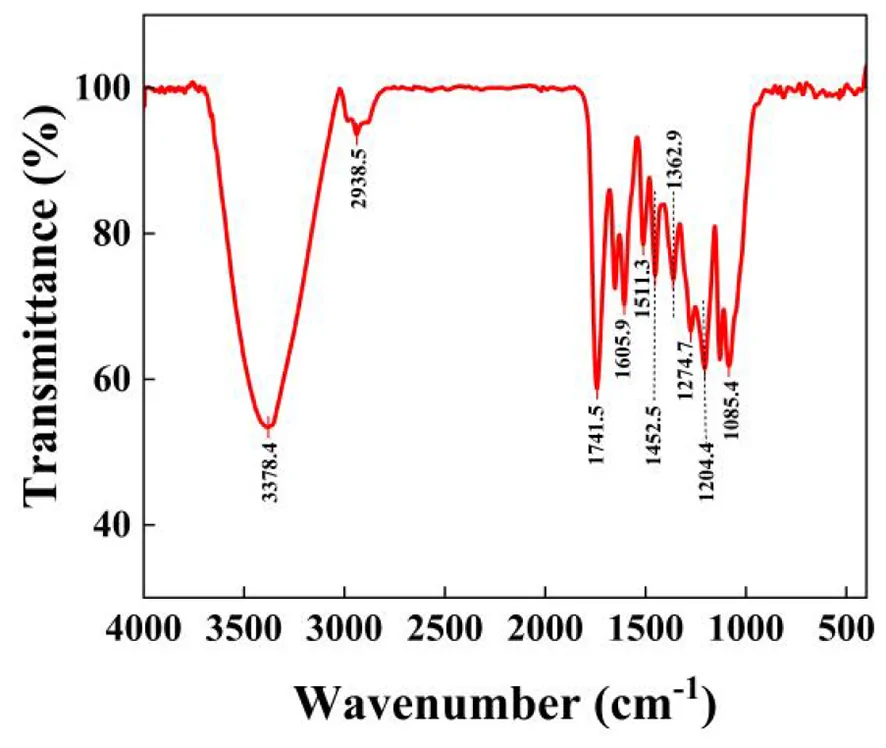
FT-IR spectrum of purified peach leaf polyphenols.

The FTIR spectrum obtained for the peach leaf polyphenols following purification with AB-8 macroporous resin is presented in Figure 6. A prominent, broad absorption maximum appearing at 3378.4 cm^−^^1^ was assigned to the stretching vibration of O–H groups, indicating the presence of abundant hydroxyl groups that are characteristic of plant polyphenols. The signal detected at 2938.5 cm^−^^1^ arose from aliphatic C–H stretching (37, 38). Another distinct absorption band at 1741.5 cm^−^^1^ was assigned to the stretching vibration of carbonyl (C = O) groups, which may originate from esterified or carboxyl-containing compounds commonly found in plant phenolics. The absorptions at 1605.9 and 1511.3 cm^−^^1^ corresponded to aromatic C = C skeletal vibrations, which are characteristic of phenolic structures. Moreover, the peaks positioned at 1274.7, 1204.4, and 1085.4 cm^−^^1^ were consistent with C–O and C–O–C stretching modes, typically associated with phenolic hydroxyls, aromatic ether linkages, and glycosidic moieties (39).

Overall, the FTIR spectrum exhibited the characteristic functional groups commonly observed in plant-derived polyphenols, including phenolic hydroxyl groups, aromatic rings, carbonyl groups, and glycosidic linkages. These spectral features are consistent with those reported for flavonoid-rich plant extracts, supporting the successful enrichment of polyphenolic constituents after AB-8 macroporous resin purification and providing structural evidence for the subsequent compositional characterization and antioxidant activity evaluation.

#### 3.4.2 UPLC–MS/MS analysis of peach leaf polyphenols

**Figure 7:**
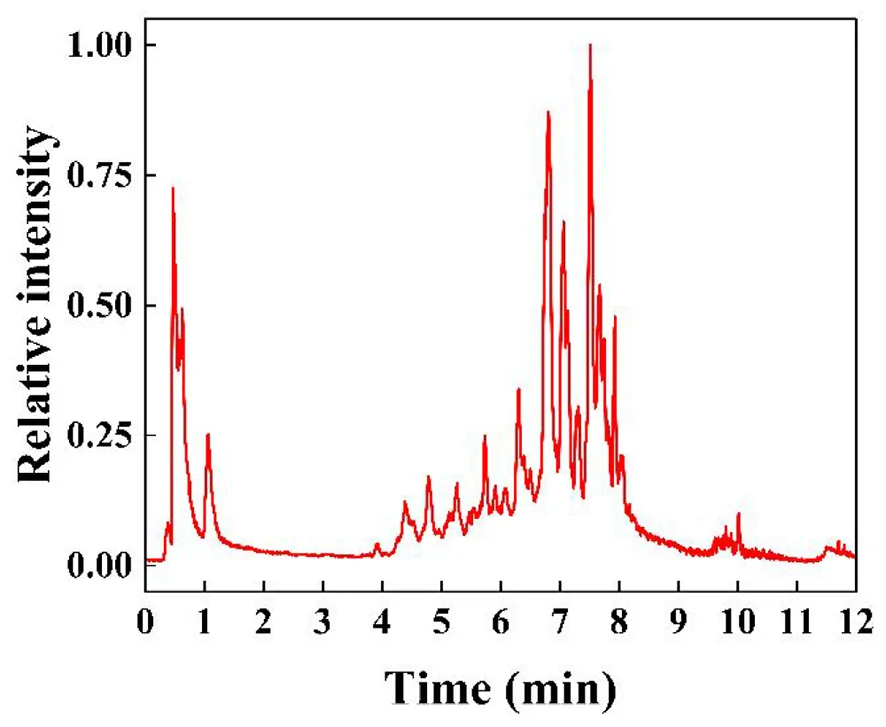
Total ion chromatogram (TIC) of peach leaf polyphenols obtained by UPLC–MS/MS.

After purification using AB-8 macroporous adsorption resin, the polyphenol purity reached 59.65 ± 0.62%. This result indicated that the resin effectively enriched phenolic compounds while reducing the interference of non-phenolic substances, thereby providing a high-quality sample for subsequent UPLC–MS/MS characterization. The phenolic composition of the purified extract was characterized by UPLC–MS/MS. Based on the retention times, precursor/product ion information, and comparisons with authentic standards, a total of 25 phenolic compounds were identified (Figure 7 and Table 4). These compounds were classified into flavonol glycosides, flavonols, dihydroflavonols, flavanols, hydroxybenzoic acid derivatives, cinnamic acid derivatives, and flavones. Among them, flavonol glycosides were the predominant constituents, accounting for 91.07% of the total quantified polyphenols, indicating that the purified extract was highly enriched in flavonol glycosides.

**Table 4:** Classification and relative abundance of phenolic compounds identified in purified peach leaf polyphenols by UPLC–MS/MS.

Chemical class	Representative compounds	Compound proportion (%)	Class proportion (%)
Flavonol glycosides	Quercetin-3-*O*-glucosid,	50.75	91.07
	Kaempferol-3-*O*-glucoside	20.71	
	Rutin	19.61	
Flavonols	Quercetin	1.80	1.92
	Kaempferol	0.12	
Dihydroflavonols	Dihydromyricetin	1.80	1.96
	Dihydroquercetin	0.12	
	Dihydrokaempferol	0.04	
Flavanols	Catechin	0.13	0.19
	Epicatechin	0.06	
Hydroxybenzoic acids and derivatives	Protocatechualdehyde	2.83	3.94
	Benzoic acid	0.58	
	3,4-Dihydroxybenzoic acid	0.45	
	Vanillic acid	0.02	
	Syringic acid	0.02	
	alicylic acid	0.03	
	Gallic acid	0.01	
Cinnamic acid derivatives	Trans-Ferulic acid	0.03	0.82
	Vanillin	0.41	
	Syringaldehyde	0.12	
	Hydrocinnamic acid	0.01	
	Caffeic acid	0.21	
	p-Hydroxycinnamic Acid	0.04	
Flavones	Vitexin	0.01	< 0.04
	Luteolin	0.03	

Quercetin-3-O-glucoside was the most abundant compound, accounting for 50.75% of the total quantified polyphenols, followed by kaempferol-3-O-glucoside (20.71%) and rutin (19.61%). Collectively, these three flavonol glycosides represented more than 90% of the quantified phenolic compounds, demonstrating that they constitute the dominant phenolic components of peach leaves. In addition, several minor constituents, including protocatechualdehyde, quercetin, dihydromyricetin, benzoic acid, protocatechuic acid, vanillin, and caffeic acid, were also detected, although their concentrations were considerably lower than those of the flavonol glycosides.

Previous studies have demonstrated that the leaves of *Prunus persica* are characterized by a phenolic profile dominated by flavonol glycosides, particularly quercetin and kaempferol glycosides, whereas phenolic acids generally occur at relatively low levels. The compositional profile obtained in the present study is in good agreement with these reports, further confirming that flavonol glycosides are the predominant phenolic constituents of peach leaves. Kim et al. (40) reported that kaempferol glycosides and quercetin glycosides were the predominant phenolic compounds in peach leaves, with the leaves exhibiting substantially higher total phenolic contents than the fruits. Similarly, Mokrani et al. (41) demonstrated that quercetin glycosides, kaempferol glycosides, and rutin were the major flavonoids in peach leaves, and their abundance was positively correlated with antioxidant activity. Although the dominant phenolic compounds identified in the present study were generally consistent with previous reports, quantitative differences were observed, which may be attributed to variations in cultivar, growing conditions, harvest season, and extraction and purification procedures. The predominance of flavonol glycosides identified in the purified extract is also consistent with the FTIR results, which revealed characteristic aromatic and glycosidic structural features. Moreover, these compounds are widely recognized for their potent antioxidant properties, suggesting that they are likely to contribute substantially to the antioxidant activity of the purified peach leaf polyphenols observed in the subsequent assays.

### 3.5 Antioxidant activity of purified peach leaf polyphenols

**Figure 8:**
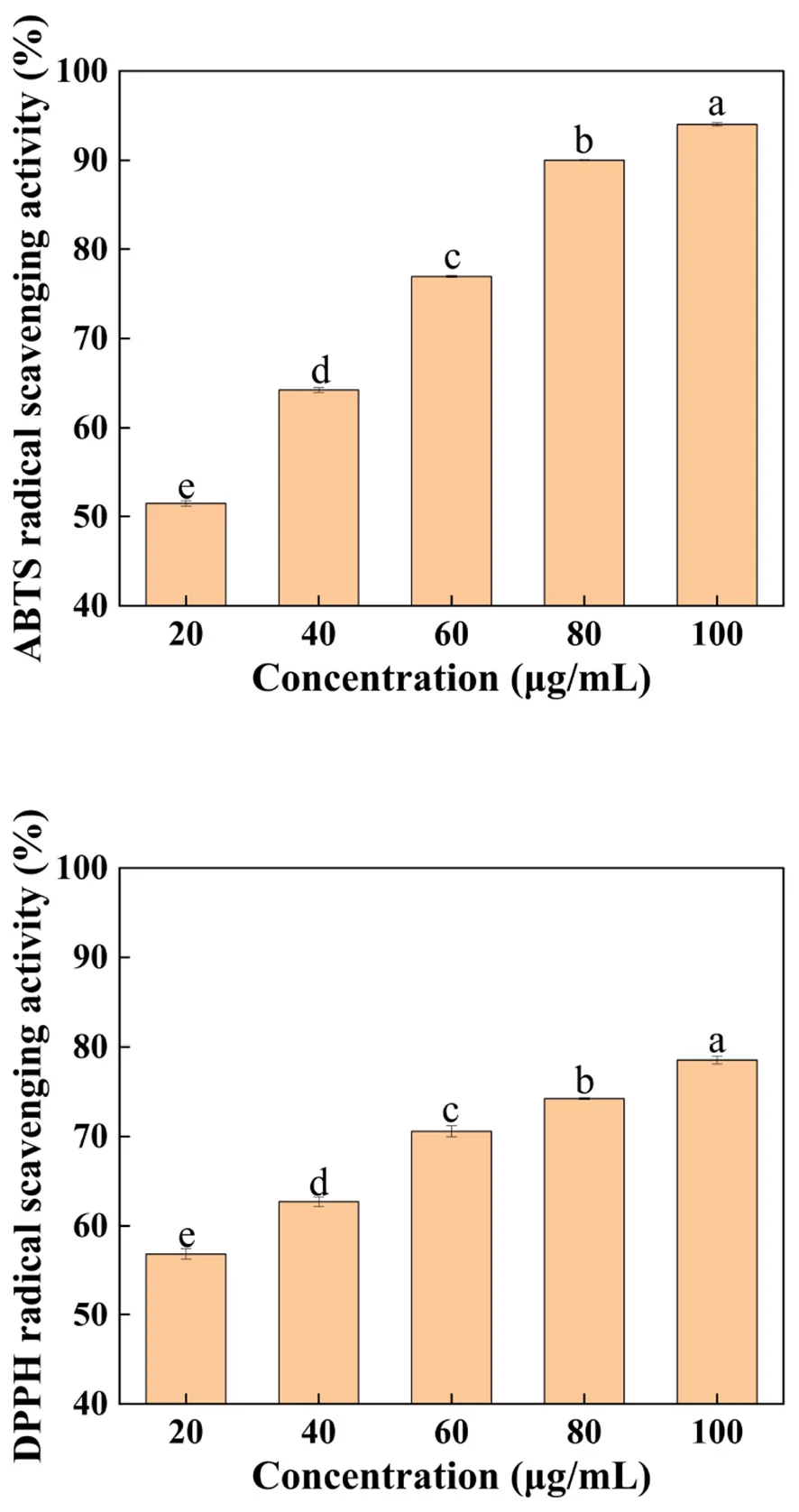
ABTS^+^ and DPPH radical scavenging activities of peach leaf polyphenols at different concentrations. Values are expressed as mean ± standard deviation (SD) (*n* = 3). Different lowercase letters indicate significant differences among treatments (*p* < 0.05).

The ABTS^+^ and DPPH radical scavenging assays are widely used *in vitro* methods for evaluating the antioxidant capacity of natural products, as they reflect the ability of antioxidants to donate electrons and/or hydrogen atoms for free radical neutralization (42). As shown in Figure 8, the purified peach leaf polyphenols exhibited significant concentration-dependent scavenging activities against both ABTS^+^ and DPPH radicals (*p* < 0.05). Over the concentration range of 20–100 μg/mL, the scavenging activity toward ABTS^+^ radicals rose from 51.58% to 94.03%, whereas that toward DPPH radicals increased from 56.86% to 78.53%. The purified extract exhibited consistently stronger scavenging activity toward ABTS^+^ radicals than toward DPPH radicals over the tested concentration range, indicating a high capacity for electron and hydrogen donation. The difference in scavenging efficiency between the two assay systems may also be related to their distinct reaction mechanisms and solvent environments, as ABTS^+^ radicals are generally more accessible to both hydrophilic and lipophilic antioxidants, whereas DPPH radicals preferentially react with compounds that are soluble in organic media. A strong correlation was observed between the antioxidant capacity of the purified extract and its phenolic profile. As demonstrated by the FTIR and UPLC–MS/MS analyses, the extract was highly enriched in flavonol glycosides, particularly quercetin-3-O-glucoside, kaempferol-3-O-glucoside, and rutin, which together accounted for more than 90% of the quantified polyphenols. These flavonoids possess multiple phenolic hydroxyl groups and conjugated aromatic structures that facilitate electron or hydrogen atom transfer, thereby effectively stabilizing free radicals. Phenolic compounds, particularly flavonoids containing multiple hydroxyl groups, have been reported to exhibit strong antioxidant properties through electron transfer and hydrogen atom donation mechanisms (43). Furthermore, purification using AB-8 macroporous adsorption resin enriched these bioactive phenolic constituents, which likely contributed to the enhanced antioxidant activity observed *in vitro*. Overall, the purified peach leaf polyphenols exhibited excellent free radical scavenging capacity, highlighting their potential as natural antioxidants and promising functional ingredients for food applications.

## 4 Conclusions

In the present work, an eco-friendly and efficient strategy was developed for the recovery of polyphenols from peach leaves by integrating ultrasound-assisted deep eutectic solvent (UAE-DES) extraction with AB-8 macroporous resin purification. Using the choline chloride–lactic acid DES system, the extraction process was optimized with the following conditions: liquid-to-solid ratio of 26.61 mL/g, ultrasonic temperature of 40 °C, choline chloride-to-lactic acid molar ratio of 1:3.715, water content of 20%, and sonication time of 10 min. Under these optimized parameters, the obtained TPC value reached 16.79 mg GAE/g, which was consistent with the predicted value from the response surface model. FTIR analysis verified that the purified material exhibited the typical spectral signatures associated with plant-derived phenolic compounds. UPLC–MS/MS profiling identified 25 phenolic constituents, with flavonol glycosides accounting for 91.07% of the quantified polyphenols. Quercetin-3-O-glucoside, kaempferol-3-O-glucoside, and rutin emerged as the major phenolic constituents. In addition, the purified extract exhibited potent concentration-dependent free radical-scavenging activity toward both DPPH and ABTS^+^ radicals, indicating strong *in vitro* antioxidant potential. Collectively, the combination of UAE-DES extraction with macroporous resin treatment offers a viable and environmentally benign pathway for enriching polyphenolic compounds from peach leaves. The obtained flavonol glycoside-rich extract shows promising potential as a natural antioxidant ingredient for functional food and related applications. This study provides a feasible approach for the preparation of peach leaf polyphenol-rich extracts and supports their potential application in the development of natural antioxidant ingredients and functional food products.

## Data Availability

The original contributions presented in the study are included in the article/supplementary material, further inquiries can be directed to the corresponding author.
